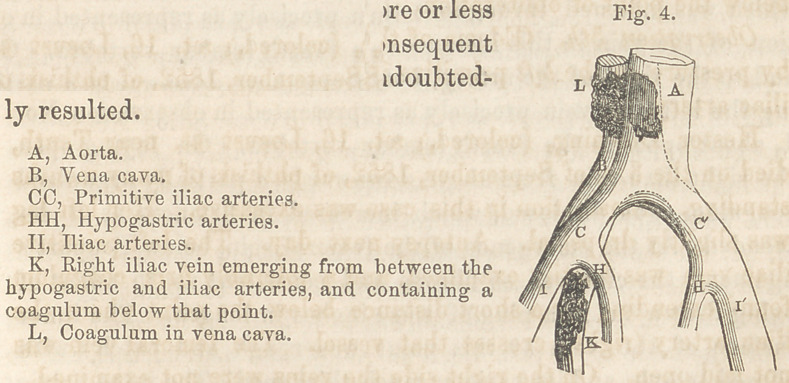# Cases of Œdema of a Single Lower Extremity

**Published:** 1856-11

**Authors:** F. W. Lewis


### *Cases of Œdema of a Single Lower Extremity.*

By F. W. Lewis, M. D.

The liability to localized serous infiltrations of various parts of the body, but more particularly of the lower extremities, so frequently observed towards the close of many chronic and wasting diseases, such as pulmonary consumption and adynamic fevers, has long been one of the recognized facts of medicine.

For the most part, pathologists have been contented to pass over in silence the study of these lesions, which, however instructive they may be to the anatomist and physiologist, really offer but little of interest in their practical bearings on the termination of disease, or its therapeutical indications; the few theories advanced to cover the obscurity of diagnosis, generally having reference to some marked condition of the blood, or to a convenient serous diathesis.

In those cases, however, where the dropsical effusion affected a single limb, usually the *left* lower extremity, no attempt had been made to assign any satisfactory cause for the phenomenon until a few years since (1843), Cruveilhier, in his Anatomy, (vol. iii, p. 86) hinted (when confined to one of the lower extremities) at the probability of a mechanical obstruction of the *right* or *left* primitive iliac vein by its corresponding artery, or by both, as the following extract will explain:—

"The relations of the primitive iliac veins to the arteries of the same name are remarkable in this, that the former lie between the arteries and the spinal column. The *right* primitive iliac vein is placed to the

*outside* and *behind* the corresponding artery, parallel to which its course lies; whereas, the *left* primitive iliac vein is situated behind and to the inside of the *left* primitive iliac artery, which latter vessel overlies it anteriorly; this same *left* primitive iliac vein, moreover, at the point where it becomes continuous with the inferior vena cava is crossed by the *right* primitive iliac artery. From this it results that the *left* primitive iliac is covered by and may be compressed by both the primitive iliac arteries, while the *right* primitive iliac vein escapes all contact with either of these vessels, and to this circumstance is probably owing the greater tendency to infiltration of the lower extremity in chronic maladies." (By a reference to plate No. 3 this will be better understood.)

In the latter part of 1847, the writer, while in Paris, enjoyed opportunities of seeing several autopsical investigations undertaken with the express view of throwing light upon the etiology of this curious phenomenon, the result of which he communicated to the Medical Examiner for May, 1848. Since that period he has been enabled to collect several other observations bearing upon the same point. These, while they serve in a measure to confirm the view then advanced, also prove the mechanical explanation to be by no means of universal application in these cases. For the satisfaction of those whose attention may not have been directed to the point in question, he will be excused for re-producing the observations contained in the former paper in a condensed form.

In many of these cases (of local dropsy) the serous effusion has been confined to a single limb, most frequently the *left* lower extremity, and to M. Piedagnel (Medicine des Hopitaux) is unquestionably due the merit of having first practically demonstrated on the dead subject that the above lesion depends on a mechanical constriction of the iliac vein, (usually the left,) by the corresponding artery, as will be sufficiently explained by the subjoined cases:

*Observation 1st.* Compression of the *left* primitive iliac vein by both the primitive iliac arteries.

The subject of this observation, a woman, æt. 50, was admitted about six weeks into the hospital (Notre Dame de la Pitié, service of M. Piedagnel) suffering under phthisis, during the progress of which malady she had become frightfully emaciated. She died Nov. 13th, 1847, and an autopsy was made on the same day. For some time previous to her decease

M. Piedagnel had directed our attention to the fact that the left leg was the seat of a considerable serous inflammation, no analogous condition being observable in the right leg, or in any other part of the body. Of this phenomenon he offered the following explanation: "When," said he, "during the progress of any wasting disease, there is great emaciation, the fat which surrounds the aorta and its primitive divisions having become absorbed, the primitive iliac vein (left), which has previously been separated from the vertebral column by an interposed layer of adipose tissue, is brought into direct contact with the bony structure of the bodies of the third or fourth lumbar vertebræ, against which it is forcibly compressed by the primitive iliac arteries, one or both; this results from the peculiar anatomical relations of the veins and arteries (iliac) to each other."

As M. Piedagnel had anticipated in this case, the left primitive iliac vein was found to be forcibly compressed by both the primitive iliac arteries, and immediately below the point of compression existed a consistent semi-organized coagulum, extending as far down as the beginning of the femoral vein. There were, moreover, evident traces of inflammatory action as evidenced by lymphous adhesion, and roughened deposits on the inner coat of the vessel.

The veins of the right side (iliac) contained little or no blood. The disposition of the arteries and veins relatively to each other and to the vertebral column is shown in the subjoined cut.

In this case the division of the aorta into its primitive iliacs, took place immediately opposite the third lumbar vertebra, and the position of the vena cava was considerably farther to the right than is usually the case.

Fig. 1.

A, Aorta.

B, Vena cava.

CC, Right and left primitive iliac arteries.

DD, " " " veins.

EE, Hypogastric arteries.

F, Point of compression.

*Observation 2d.* Œdema of the *left* lower extremity, due to compression of the *left* iliac vein by its corresponding artery.

A woman having died of phthisis (at la Pitié Hospital), an autopsy was made on the morning of the 30th of November, 1847. A dropsical condition of the left leg, similar to that observed in the former case, existed for some time before the patient's death, and the emaciation, as in that instance, was very considerable. A slight anatomical difference in the arrangement of the vessel was remarked between this and the subject of the preceding observations, owing to which the right iliac artery did not as in that instance participate in causing the pressure. The left iliac artery, in crossing, strongly compressed the iliac vein of the same side against the vertebral column. The latter vessel, on being laid open, was found to be filled with very dark coagula, more or less firm and evidently of long formation. These were in places adherent to the parietes of the vein, which gave evidence of having been inflamed. Similar adhesions, together with coloration of the serous lining, extended along the femoral vein almost to the termination. On the right side the veins were free from blood, comparatively speaking.

Fig. 2.

A, Aorta.

B, Vena cava.

CC, Right and left iliac arteries.

DD, " " veins.

F, Coagulum.

*Observation 3d.* Compression of the *left* femoral vein by a mass of indurated ganglions, with anomalous relations of the pelvic veins and arteries.

A man, æt. 88, was admitted into the Hospital la Pitié, service of M. Piedagnel, in October, 1847, affected with a chronic dysentery, contracted on the island of Martinique, during the course of which disease he emaciated very rapidly, and to such an extent that his coxal bones nearly protruded from the skin; his body much resembling a skeleton covered with parchment, with the exception of the left lower extremity, which presented a remarkable difference in this respect, being the seat of dropsical infiltration, and unusually plump and rounded. He died on the

10th December. M. Piedagnel judged that in this case, as in the two preceding, a compression of the left iliac vein by the corresponding artery would be found to exist. Such, however, did not prove to be the fact, the relation of the arteries to the veins being such that the *left* primitive iliac vein was abruptly crossed opposite the last lumbar vertebra by the hypogastric artery; but in no part of its course was this vein traversed by the primitive iliac artery of the same side. The artery was in this instance considerably in advance of the vein and absolutely parallel with it. But in crossing the *left* iliac vein, the hypogastric artery finding no *point d'appui* against the vertebral column, no compression sufficiently powerful to interfere with a due return of the venous blood to the vena cava, was exercised on the former vessel. In the femoral vein of the same side, however, at a point immediately above where that vessel emerges from beneath Poupart's ligament, was found the seat of obstruction, due to the pressure of several indurated inguinal ganglions directly upon the vein, giving rise to stases of blood in the corresponding limb. See sketch.

Fig. 3.

A, Aorta.

B, Vena cava.

CC, Primitive iliac arteries.

DD, " veins.

H, Hypogastric artery.

*Observation 4th.* Œdema of the *left* lower extremity, caused by obstruction of the left primitive iliac vein by pressure of a mass of indurated ganglions within the pelvis.

Robert Mayberry, æt. 48, No. 14 Adams st., died on April 12th, 1854, from the combined effects of chronic dysentery contracted in Mexico, of diabetes and of phthisis. He had been very intemperate, both in eating and drinking. For several weeks before death the left leg was observed to be largely infiltrated and very sensitive to the touch. At the same time the patient complained of a deep seated pulsative pain, pointing from the small of the back towards the left groin. He became very much emaciated towards the last, finally sinking from œdema of the glottis.

Autopsy on the 13th. Both kidneys were slightly enlarged, flabby and congested with dark blood. The mesenteric glands were much hypertrophied and indurated, in some places being glued together by plastic exudation into large, roughened tuberculated masses. One of the largest of these lay immediately over the bifurcation of the abdominal aorta, and was firmly bound down to the spine by lymphous bands. On dissecting this away, both primitive iliac arteries together with the *left* primitive iliac vein were found to be so intimately connected with the mass as to render isolation impossible. All appearance of the vein was lost above its hypogastric branch.

The *left* crural vein was then laid open from below upwards. It presented no traces of inflammation, nor were any clots discoverable. The *left* iliac vein was in like manner free from all coagula ; the blood contained in both being thick, dark and of a tarry consistence. A slight dilatation with thickening of the coats of the vein existed below the point where obliteration occurred. The blood in the *right* vein was about of the same consistence and color as that found in the *left*, and the inner surface of both vessels intensely stained.

The cause of obstruction was here obvious; the only remarkable feature in the case being the absence of coagulum in the vein below the point of obliteration.

*Observation 5th.* Œdema of the *left* lower extremity, caused by pressure on the *left* primitive iliac vein by the *right* primitive iliac artery.

Hester Downing, (colored,) æt. 16, Locust st. near Tenth, died on the 31st of September, 1852, of phthisis of many months standing. Emaciation in this case was excessive. Her left leg was slightly dropsical. Autopsy next day. The left primitive iliac vein was hastily examined, and a tolerably firm coagulum found extending for a short distance below the point where the iliac artery (right) crosses that vessel. The femoral vein was not laid open. On the right side the veins were not examined.

In the three remaining *Observations* the *right* leg was the seat of dropsical infiltration.

*Observation 6th.* Infiltration of the *right* leg, due partly to compression by indurated inguinal glands, partly to a *strangling* of the right iliac vein by the right iliac and hypogastric arteries.

This patient, a man, æt. 40, died of a long protracted phthisical affection, for which he had been admitted into the Hospital de la Pitié some weeks before—early in December, 1847.

Unlike the previous cases, for some time before his death, the *right* leg was observed to be infiltrated. At the autopsy, indurated ganglions (inguinal) were found surrounding the origin of the right femoral vein, below which point fibrinous coagula of recent formation obstructed the vessel, whose internal surface was rough and unpolished, and in some places deeply stained. Another coagulum of considerable density and apparently long formed, existed in the origin of the vena cava at about an inch above the division of the aorta, resting in a kind of aneurismal sac, constituted by a dilatation of the vein. Above this point, up to which the primitive iliac freely carried its blood, no obstruction could be detected, nor could the presence of the clot be accounted for. A third coagulum, also of ancient formation, filled up the right iliac vein, at a point intermediate between its origin and its termination in the vena cava. In this instance, pursuing a serpentine course, the vein passing at first from right to left and from behind forward, wound its way between the right iliac and hypogastric arteries, in the manner which the rough sketch below serves to illustrate. From these unnatural relations of the right iliac vein, more or less strangling of that vessel and consequent obstruction of the circulation undoubtedly resulted.

Fig. 4.

A, Aorta.

B, Vena cava.

CC, Primitive iliac arteries.

HH, Hypogastric arteries.

II, Iliac arteries.

K, Right iliac vein emerging from between the hypogastric and iliac arteries, and containing a coagulum below that point.

L, Coagulum in vena cava.

*Observation 7th.* Œdema of the *right* lower extremity, shortly followed by a similar condition of the left, probably caused by the decubitus of the patient, together with great torpor of circulation.

Margaret Wiley, æt. 37, No. 7 Lombard st., died the 22d of

January, 1851, of phthisis, the termination being hastened by an attack of acute dysentery. There had been nothing remarkable in the history of this case. Emaciation commenced early, and progressed rapidly, and extreme dyspnœa had always been a prominent symptom. When I first saw her, three weeks before her death, she complained of great difficulty of breathing, together with a deep seated gravitative sense of uneasiness, which she referred to the small of the back, and which could only be relieved by her lying upon the right side with the knees and thighs strongly flexed. She was fearfully wasted, with the exception of the right leg, which was very much swollen, pitting deeply on pressure. The heart's action was feeble, and intermitting.

About a week before her death, owing to orthopnœa and extreme debility, she was forced to lie upon her back always, with the thighs strongly flexed on the pelvis; and within a short time after this change of posture, infiltration of the left leg was observed to commence. This had not advanced to any very great extent when she died.

Autopsy on the 23d. On opening the abdomen the contained viscera were healthy, with the exception of the pancreas, which was enlarged to double the usual size, the interior being of a reddish chocolate color and much softened, and the large intestine which was inflamed throughout the entire length. The bifurcation of the vein in this instance took place considerably above, and to the right of the artery, both the right and left iliac crossing the left iliac vein precisely as represented in observation No. 1, about opposite the fourth lumbar vertebra. On laying open the right iliac vein from above downwards, a partially organized coagulum was disclosed, which increased in firmness as it descended. At the emergence of the femoral vein, below Poupart's ligament, the clot was so fibrous and consistent as to admit of being drawn out in a plug of some inches in length. The inner surface of the vein at this point was rough and patchy with effused lymph, and its parietes much thickened. The intra pelvic portion of the vein presented these phenomena in a slight degree only. In the *left* iliac vein a clot of comparatively recent formation obstructed the calibre of the vessel. In the crural vein this coagulum was relatively more dense. No perceptible enlargement of the inguinal glands existed.

In this case it will be remarked that all the conditions present were similar to those observed in the subject of observation No. 1. These, viz.: the excessive emaciation, the oblique crossing of the left iliac vein by both iliac arteries, at the point of greatest prominence of the lumbar vertebræ, (opposite the body of the 4th) should, according to M. Piedagnel, inevitably have resulted in the production of a coagulum of greater or less density in the *left* iliac vein. That no such coagulum did form under these favorable circumstances is not more remarkable, however, than that a clot should be found in the right femoral vein, and subsequently in the corresponding vein of the left side, at a point where no apparent interruption to the circulation or obliteration of the calibre of the vessel could be detected in either case. The only explanation of these phenomena is suggested by the constrained and unnatural decubitus of the patient for the last few weeks of her illness, viz.: on the right side with the thighs and knees strongly flexed. It is conceivable that this position, by throwing the superposed weight of half the body upon the right side, and by the constant flexion of the vein, might, in a feeble and torpid state of the circulation, favor a stasis of blood on the part alluded to.

*Observation 8th.* Œdema of the right leg, probably dependent on the position of the patient.

Theresa Ferris, æt. 23, Wilcox st. No. 16, died of phthisis on the 13th of September, 1856. She had been long suffering. About six weeks before death, she was brought to bed of her second child. The labor was rapid, easy and followed by no untoward symptoms. She rose on the 8th day, but was shortly obliged to return to her bed, owing to the rapid progress of her thoracic disease. About three weeks before her death the *right* leg began to infiltrate. This was three weeks after her confinement. She complained of slight pain in the limb. The decubitus of this patient was usually on the right side up to about a week before death, when it became dorsal.

*Autopsy on the next day.*—Emaciation extreme. The relative positions of both arteries and veins (iliac) were normal. On laying open the right primitive iliac vein and the right femoral vein through their entire length, their calibre was found to be obliterated by a semi-organized coagulum, extending from the bifurcation of the iliac veins continuously as far as the incision was carried, viz., the upper third of the thigh. This coagulum increased in density as it descended the vein. The parictes of the femoral vein (right) were much thickened, so much so as at first to convey the impression that the corresponding artery had been opened. The right iliac vein within the pelvis was not materially altered in this respect. No traces of lymph adhering to the inner surface of the vein, roughening of its walls, or other evidence of inflammatory action, saving the thickening of the parietes, before spoken of, were present, thus removing all suspicion of the pre-existence of post-puerperal phlebitis as a cause of the lesion in question. The *left* iliac vein contained no coagulum.

It will be remarked that in the subject of this observation, the dropsy was confined exclusively to the *right* leg, and, moreover, that the clot extended along the entire course of the vein, ceasing abruptly where that vessel joins the inferior vena cava.

What could, in this instance, have been instrumental in giving rise to obstruction of the circulation at that point? Emaciation in this case was very great, and the bony prominence formed by the bodies of the lumbar vertebræ remarkably large and resisting; the artery, (right iliac) too, overlying the *left* iliac vein in a position the most favorable for causing obstruction in the latter vessel, and yet, as in the *preceding observation*, the right iliac vein was the seat of the coagulum.

It is scarcely possible in reviewing the striking points of similarity in these *two* cases, not to admit the probable agency exercised by the *decubitus* of the patients upon the right side, in causing the lesion. How much might have been due to the pressure of the superincumbent viscera upon the vein in obliterating its calibre, can only be the merest matter of speculation.

To sum up, of the *eight* observations reported, *five* were cases of œdema of the *left* lower extremity, and *three* of œdema of the *right.*

The most common *cause* of obstruction in the former is undoubtedly of one or both iliac arteries upon the left primitive iliac vein; two cases of the five having originated in glandular pressure on the vein.

In the remaining *three* cases where the *right* leg was affected, one owed its origin to glandular obstruction, and also to compression of the *right* iliac vein by the *right* iliac and hypogastric arteries; for the two other cases no positive point of departure could be ascertained.

## Figures and Tables

**Fig. 1. f1:**
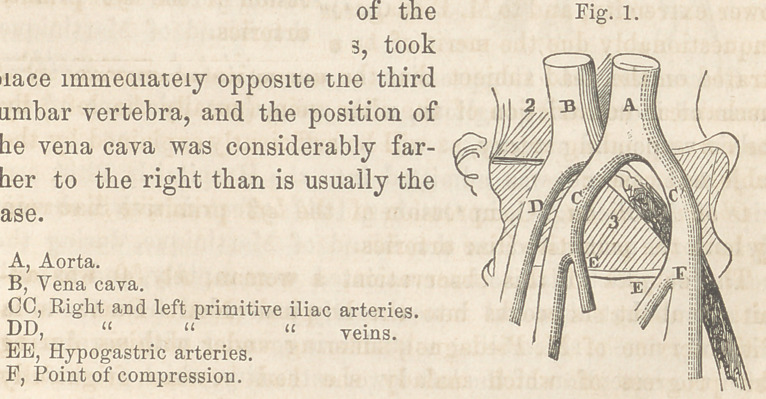


**Fig. 2. f2:**
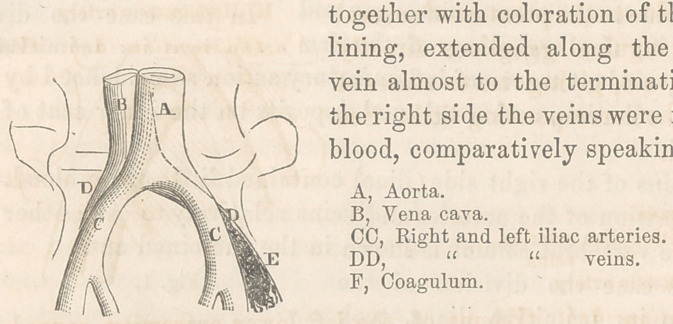


**Fig. 3. f3:**
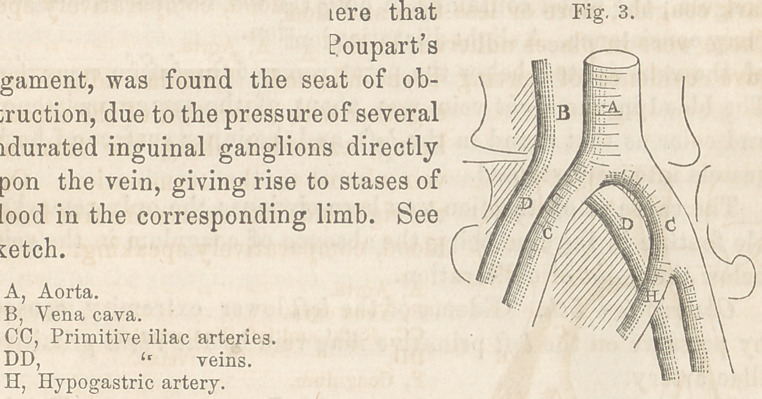


**Fig. 4. f4:**